# Supplementary material for: Amphiphilic Diblock Copolymers Bearing Poly(Ethylene Glycol) Block: Hydrodynamic Properties in Organic Solvents and Water Micellar Dispersions, Effect of Hydrophobic Block Chemistry on Dispersion Stability and Cytotoxicity
Source: Polymers (Basel). 2022 Oct 16;14(20):4361. doi: 10.3390/polym14204361 (PMC9612359; doi:10.3390/polym14204361)
Supplement: Supplementary file 1 [file polymers-14-04361-s001.zip › polymers-1925253-supplementary.pdf]

*Supplementary information for the Article:*

# Amphiphilic Diblock Copolymers Bearing Poly(ethylene glycol) Block: Hydrodynamic Properties in Organic Solvents and Water Micellar Dispersions, Effect of Hydrophobic Block Chemistry on Dispersion Stability and Cytotoxicity

**Anastasiia A. Elistratova [1], Alexander S. Gubarev [2], Alexey A. Lezov [2], Petr S. Vlasov [1], Anastasia I. Solomatina [1], Yu-Chan Liao [3], Pi-Tai Chou [3], Sergey P. Tunik [1], Pavel S. Chelushkin [1,*] and Nikolai V. Tsvetkov [2,*]**

[1] Institute of Chemistry, St. Petersburg State University, Universitetskii Av., 26, 198504 St. Petersburg, Russia
[2] Department of Molecular Biophysics and Physics of Polymers, St. Petersburg State University, Universitetskaya nab., 7/9, 199034 St. Petersburg, Russia
[3] Department of Chemistry, National Taiwan University, No. 1, Sec. 4, Roosevelt Rd., Taipei 10617, Taiwan
* Correspondence: p.chelushkin@spbu.ru (P.S.C.); n.tsvetkov@spbu.ru (N.V.T.)

## Part 1. Synthesis and characterization of block copolymers

All block copolymers used in the study were commercial. According to the manufacturers, they were prepared as follows:

PS-*b*-PEG was prepared by living anionic polymerization, with PS block being prepared first and used as a macroinitiator for subsequent ethylene oxide polymerization. PMMA-*b*-PEG was prepared using OMe-PEG-OH as a macroinitiator for PMMA polymerization according to the following scheme:

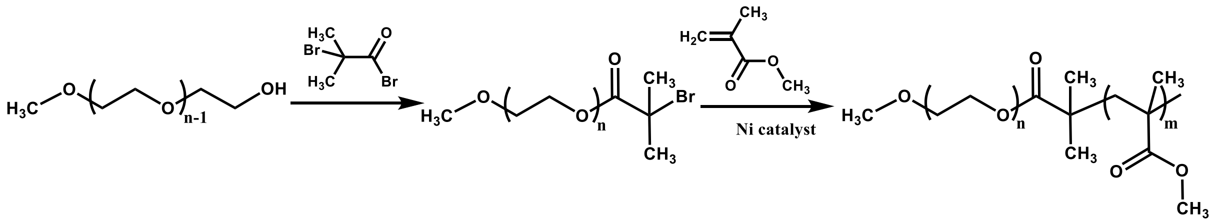


PBd-*b*-PEG was prepared by living anionic polymerization, with hydrophobic blocks being prepared first followed by ethylene oxide polymerization. PBd block microstructure is 1,4-rich but contains products of 1,2-addition. PE-*b*-PEG was prepared by hydrogenation of PBd-*b*-PEG. PDMS-*b*-PEG was prepared by living anionic polymerization of hexamethyl cyclotrisiloxane followed by hydrosililation reaction with allyl PEG using Pr catalyst according to the following scheme:

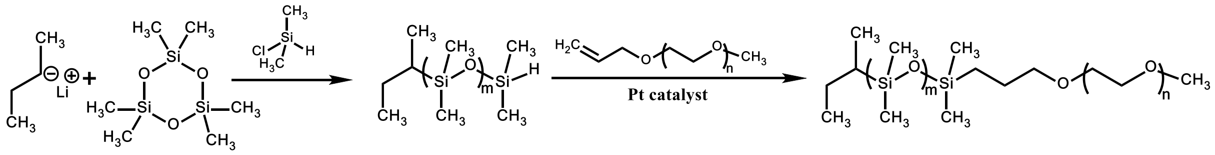


The synthetic scheme for PCL-*b*-PEG was not disclosed, but we can speculate that the block copolymer was prepared by a ring-opening polymerization of caprolactone on OMe-PEG-OH macromonomer.

Block copolymer samples were characterized by $^1$H NMR (DMSO-$d_6$) and GPC (THF, 40 °C). The degrees of polymerization (DP) listed in Table S1 in subscripts of block copolymer abbreviations were calculated based on $M_n$ values provided by the manufacturers even in the case of more or less substantial contradictions with our own experimental data. In the rest of the Supplementary Material file, due to rather similar DP for PEG and hydrophobic blocks, the DP values are omitted for simplicity.

**Table S1.** The experimentally determined hydrodynamic parameters of the solvents used in the study at corresponding temperatures.

| Solvents / Mixture (Temperature) | $\eta_0$, [cP] | $\rho_0$, [g/cm$^3$] |
|---|---|---|
| THF (20 °C) | 0.5 | 0.8882 |
| $H_2O$ (25 °C) | 0.89 | 0.9971 |
| $D_2O/H_2O$ (50/50 Vol., 25 °C) | 0.9945 | 1.0507 |

**Table S2.** Partial specific volumes and refractive index increments of the studied copolymers in THF solutions (20 °C) and the micelles in $H_2O$ solutions (25 °C) prepared with the corresponding copolymers.

| Block Copolymer | THF 20°C | | $H_2O$ 25°C | |
|---|---|---|---|---|
| | $\bar{\upsilon}$ [a] [cm$^3$/g] | d*n*/d*c* [b] [cm$^3$/g] | $\bar{\upsilon}$ [cm$^3$/g] | d*n*/d*c* [b] [cm$^3$/g] |
| PS-*b*-PEG | 0.933 | 0.126 | 0.882 [c] | 0.152 |
| PMMA-*b*-PEG | 0.857 | 0.069 | 0.814 [c] | 0.117 |
| PBd-*b*-PEG | 0.97 | 0.091 | 0.974 [c] | 0.149 |
| PE-*b*-PEG | 0.956 | 0.073 | 0.910 [a] | 0.114 |
| PDMS-*b*-PEG | 0.862 | 0.056 | 0.883 [c] | 0.072 |
| PCL-*b*-PEG | 0.908 | 0.075 | 0.879 [c] | 0.115 |

[a] Densitometry data; [b] AUC data; [c] The data are obtained with the density variation approach.

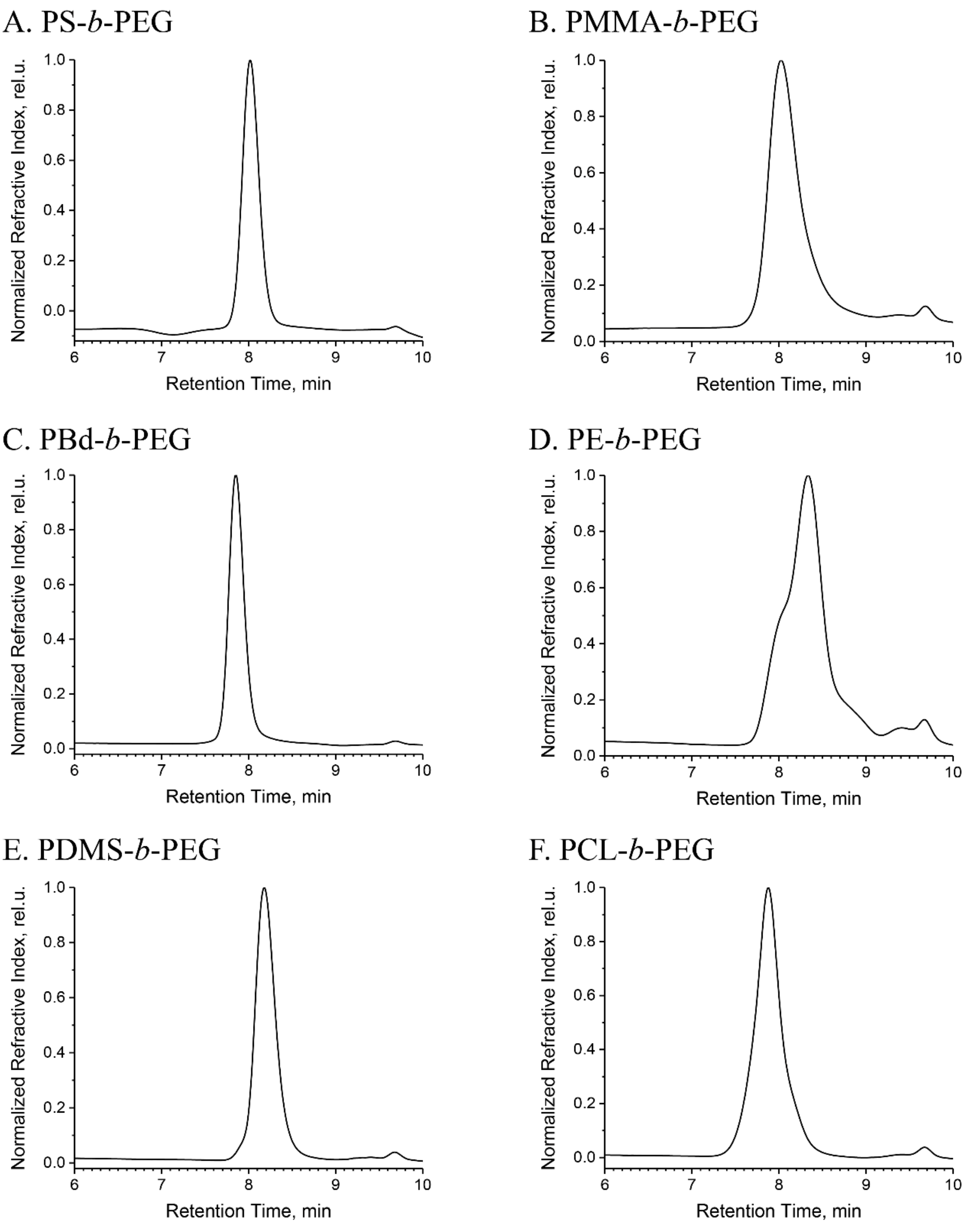


**Figure S1**. GPC traces for PS-*b*-PEG (A), PMMA-*b*-PEG (B), PBd-*b*-PEG (C), PE-*b*-PEG (D), PDMS-*b*-PEG (E), PCL-*b*-PEG (F) in THF at 40 °C.

**Part 2. Preparation and stability of block copolymer micelles in aqueous dispersions**

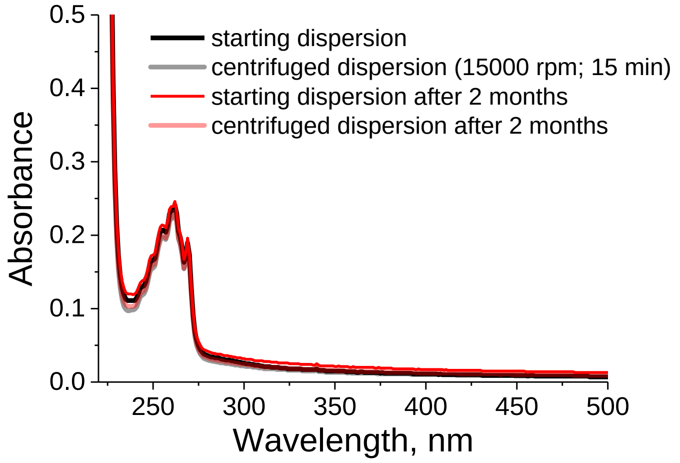


**Figure S2.** UV-vis absorption spectra for PS-*b*-PEG micelles at 0.5 mg/mL before and after centrifugation as well as after 2 months of storage.

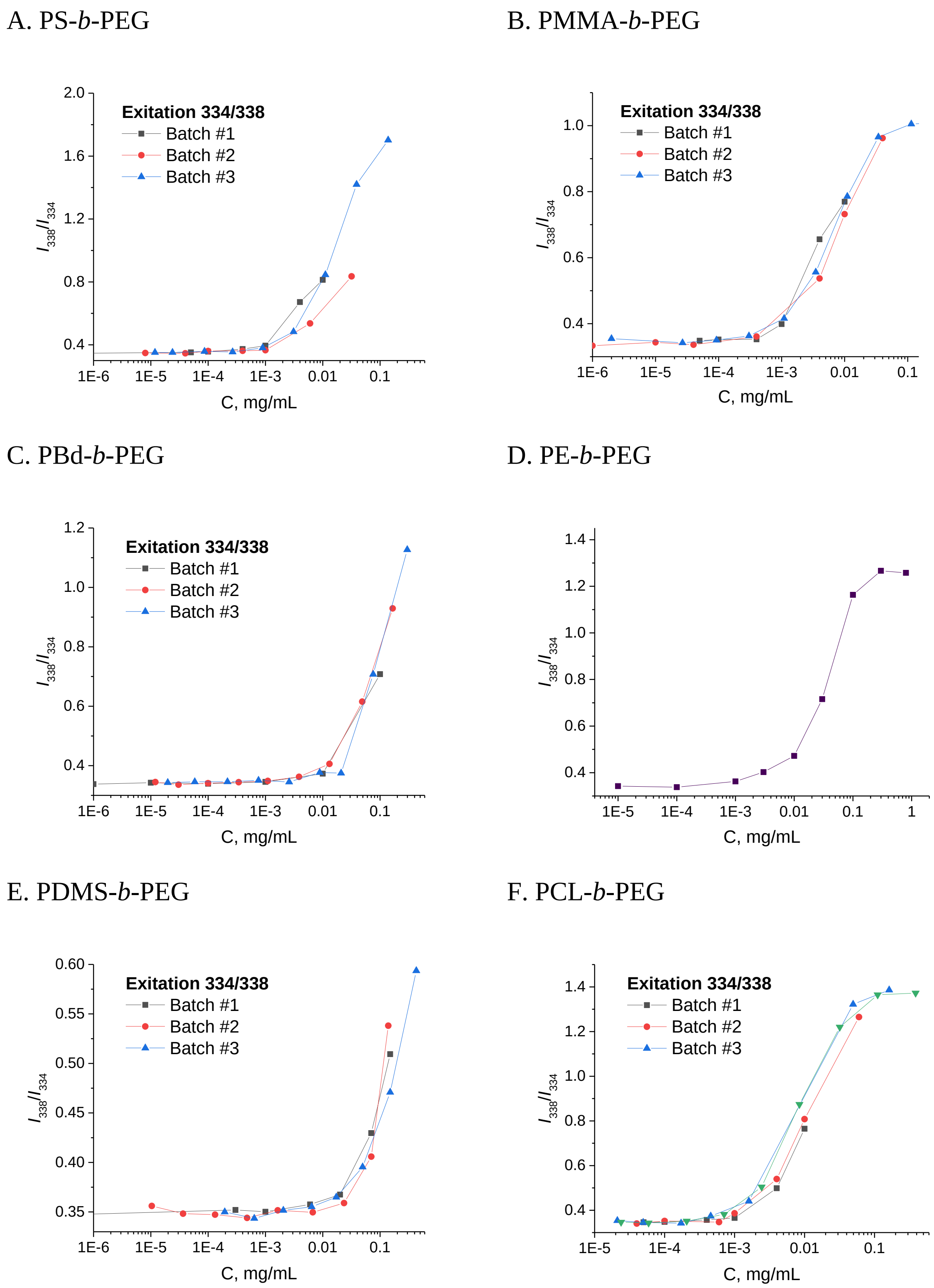


**Figure S3.** Concentration dependences of intensity ratios $I_{338}/I_{334}$ taken from excitation spectra of pyrene in aqueous micellar dispersions of PS-*b*-PEG (A), PMMA-*b*-PEG (B), PBd-*b*-PEG (C), PE-*b*-PEG (D), PDMS-*b*-PEG (E), PCL-*b*-PEG (F). [pyrene] = $6 \cdot 10^{-7}$ M.

## Part 3. Hydrodynamic studies of block copolymer micelles in aqueous dispersions

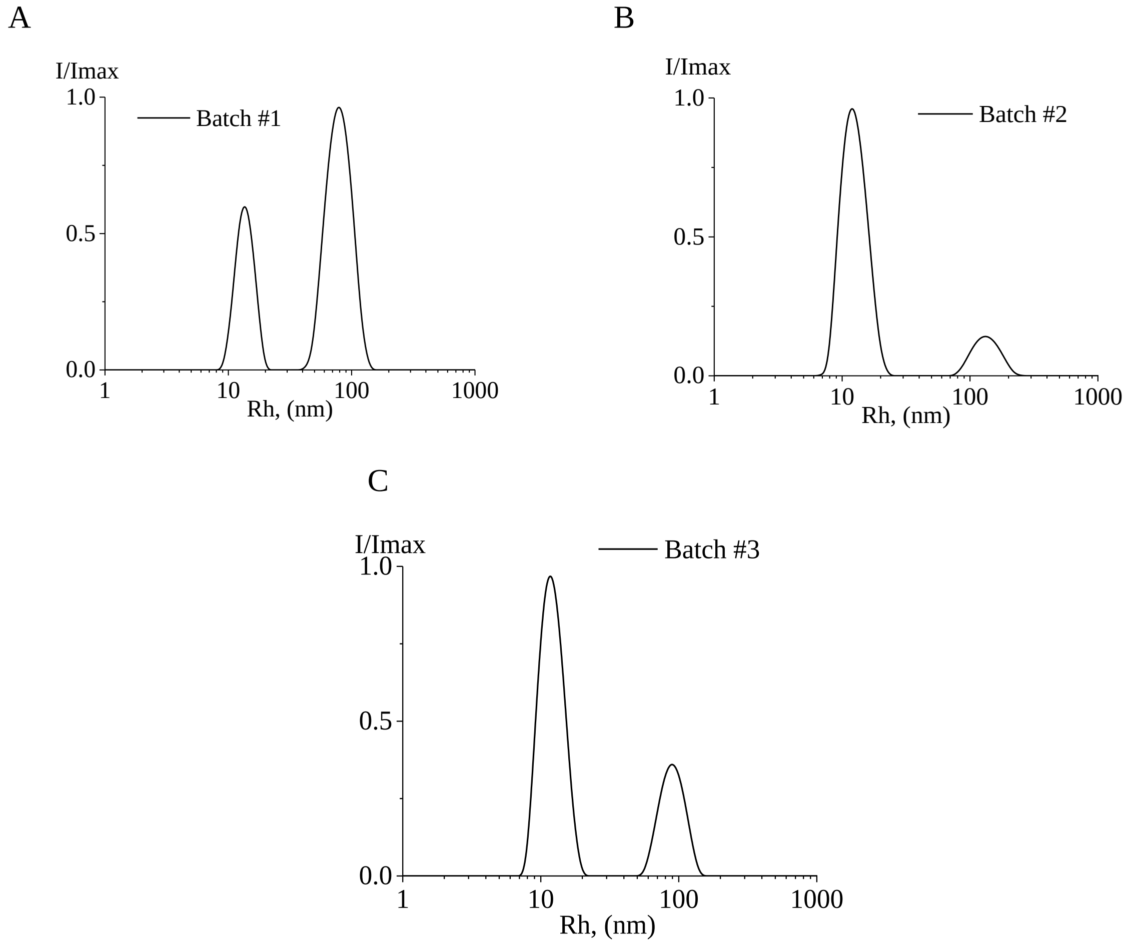


**Figure S4.** Normalized intensity weighted $R_h$ distributions for PS-*b*-PEG micelles measured for three different batches (independent micelle preparations) in water at 25 °C. Scattering angle is 90°.

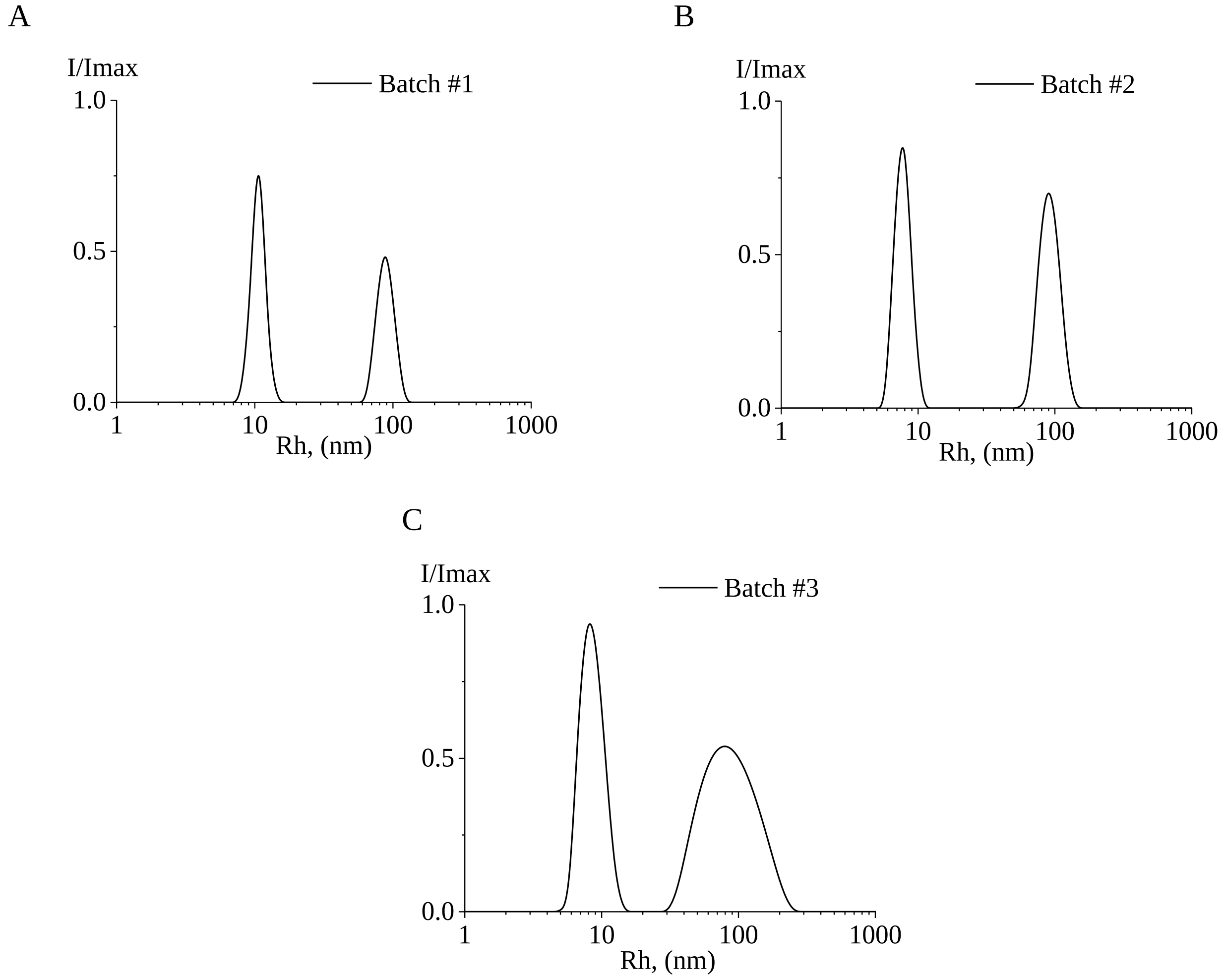


**Figure S5.** Normalized intensity weighted $R_h$ distributions for PMMA-*b*-PEG micelles measured for three different batches (independent micelle preparations) in water at 25 °C. Scattering angle is 90°.

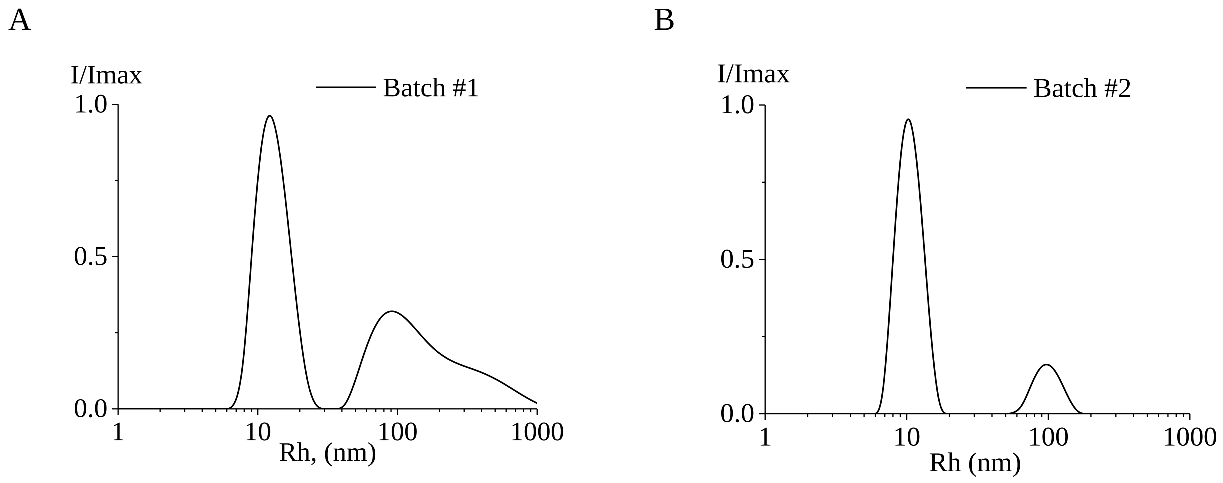


**Figure S6.** Normalized intensity weighted $R_h$ distributions for PBd-*b*-PEG micelles measured for two different batches (independent micelle preparations) in water at 25 °C. Scattering angle is 90°.

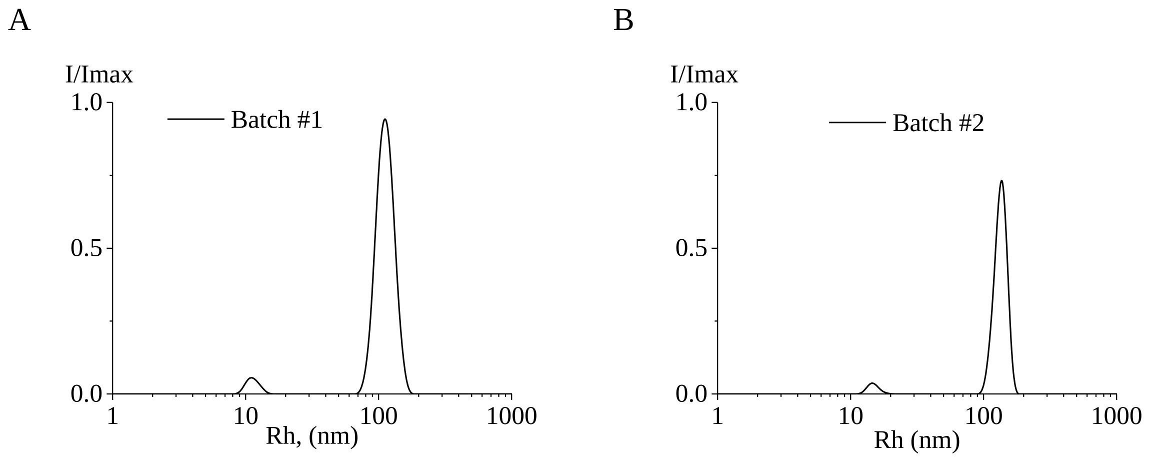


**Figure S7.** Normalized intensity weighted $R_h$ distributions for PDMS-*b*-PEG micelles measured for two different batches (independent micelle preparations) in water at 25 °C. Scattering angle is 90°.

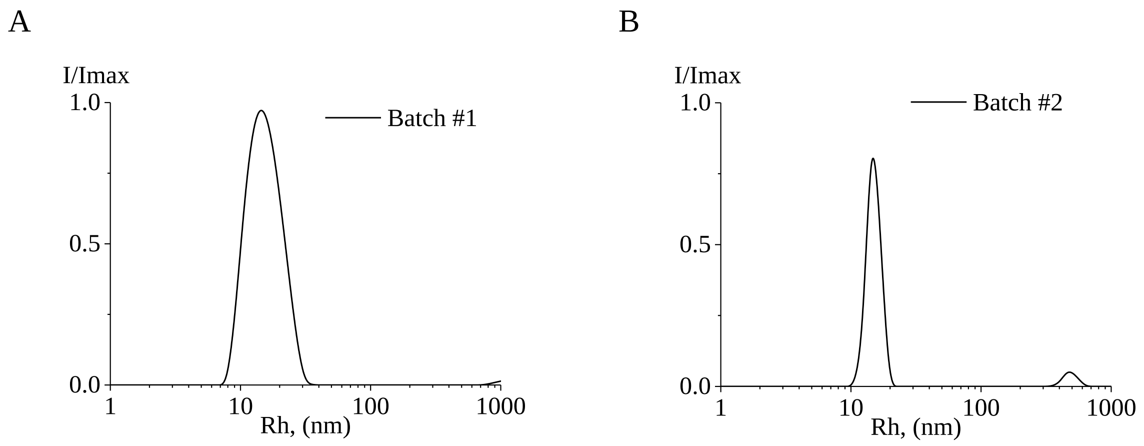


**Figure S8.** Normalized intensity weighted $R_h$ distributions for PCL-*b*-PEG micelles measured for two different batches (independent micelle preparations) in water at 25 °C. Scattering angle is 90°.

A. PS-*b*-PEG

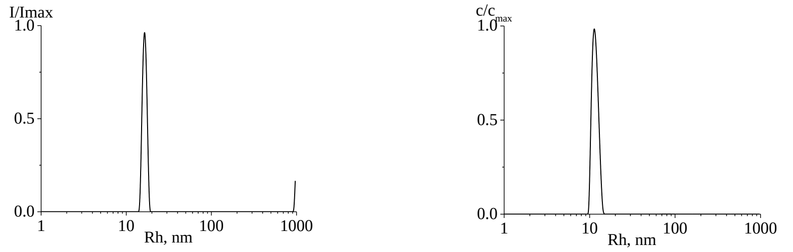


B. PMMA-*b*-PEG

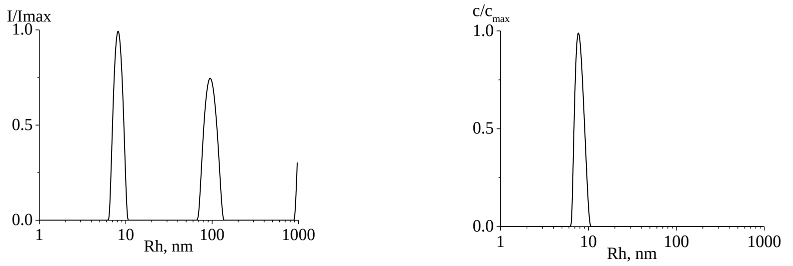


C. PBd-*b*-PEG

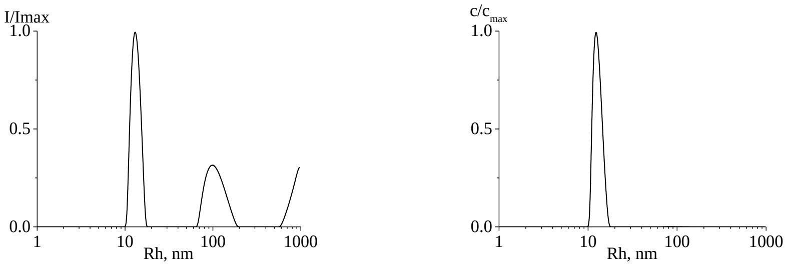


D. PE-*b*-PEG

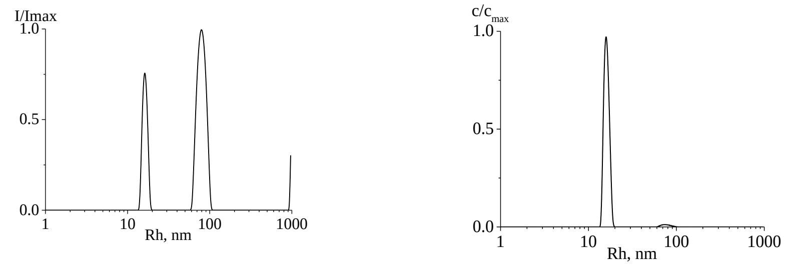


E. PDMS-*b*-PEG

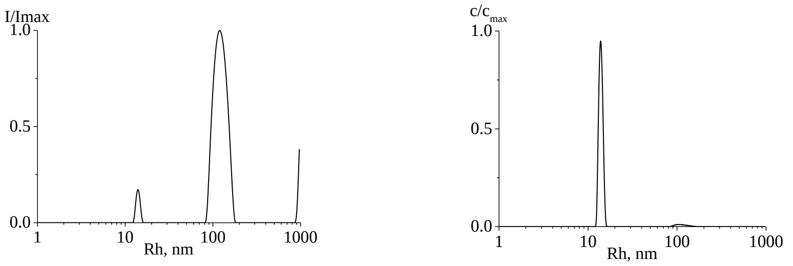


F. PCL-*b*-PEG

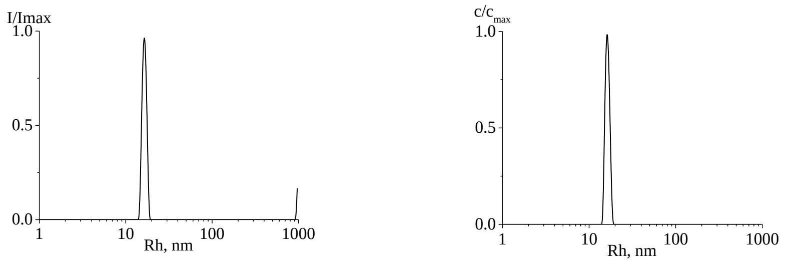


**Figure S9.** Normalized intensity weighted (left column) and mass fraction weighted (right column) $R_h$ distributions for block micelles dispersions measured in water at 25 °C. Scattering angle is 90°.

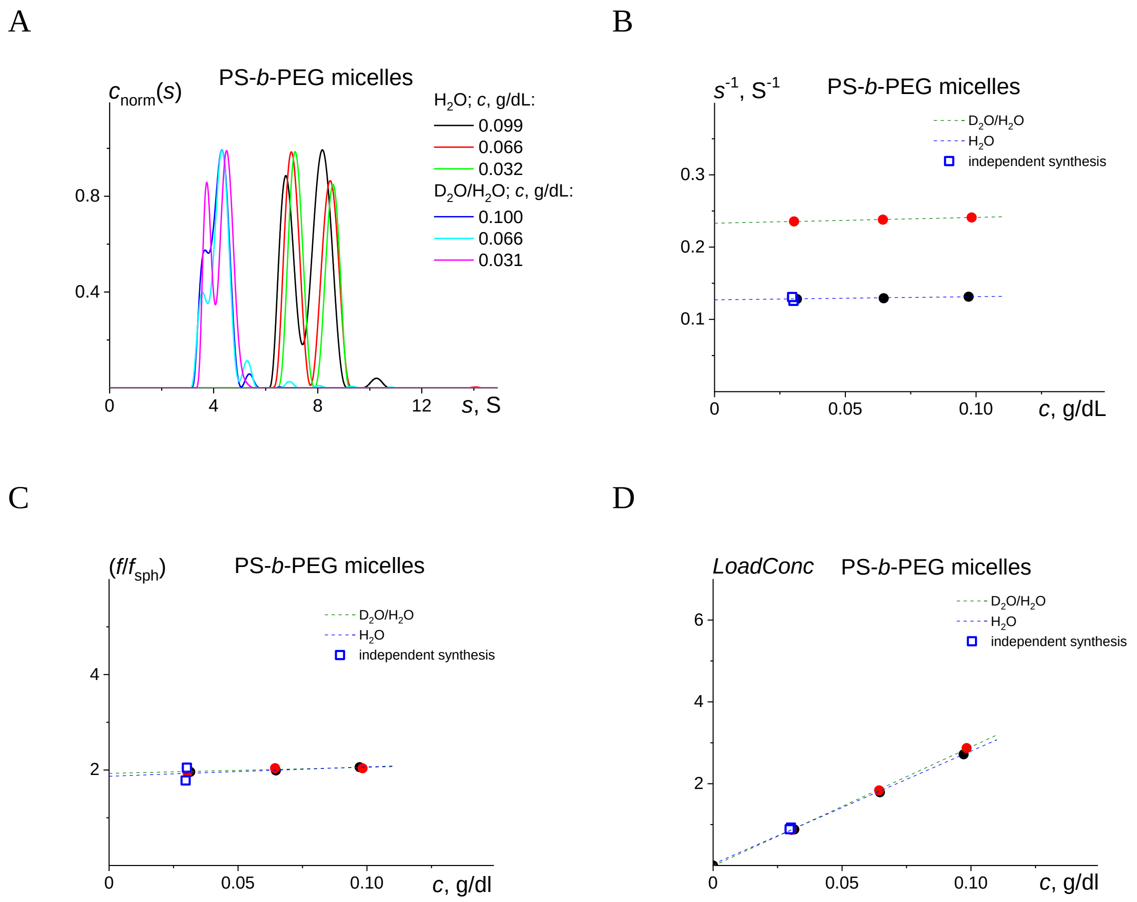


**Figure S10.** Summary of the velocity sedimentation experiment with PS-*b*-PEG block copolymer micelles in $H_2O$ and $D_2O/H_2O$ (50/50 vol.) mixture at 25 °C. (A) The normalized *c*(*s*) distributions on sedimentation coefficient *s* resolved with Sedfit ‘continuous *c*(*s*) distribution’ at three different concentrations in both solvents. (B) Concentration dependences of sedimentation coefficients. (C) Concentration dependences of the frictional ratios ($f/f_{sph}$). (D) Determination of the refractive index increment d*n*/d*c*.

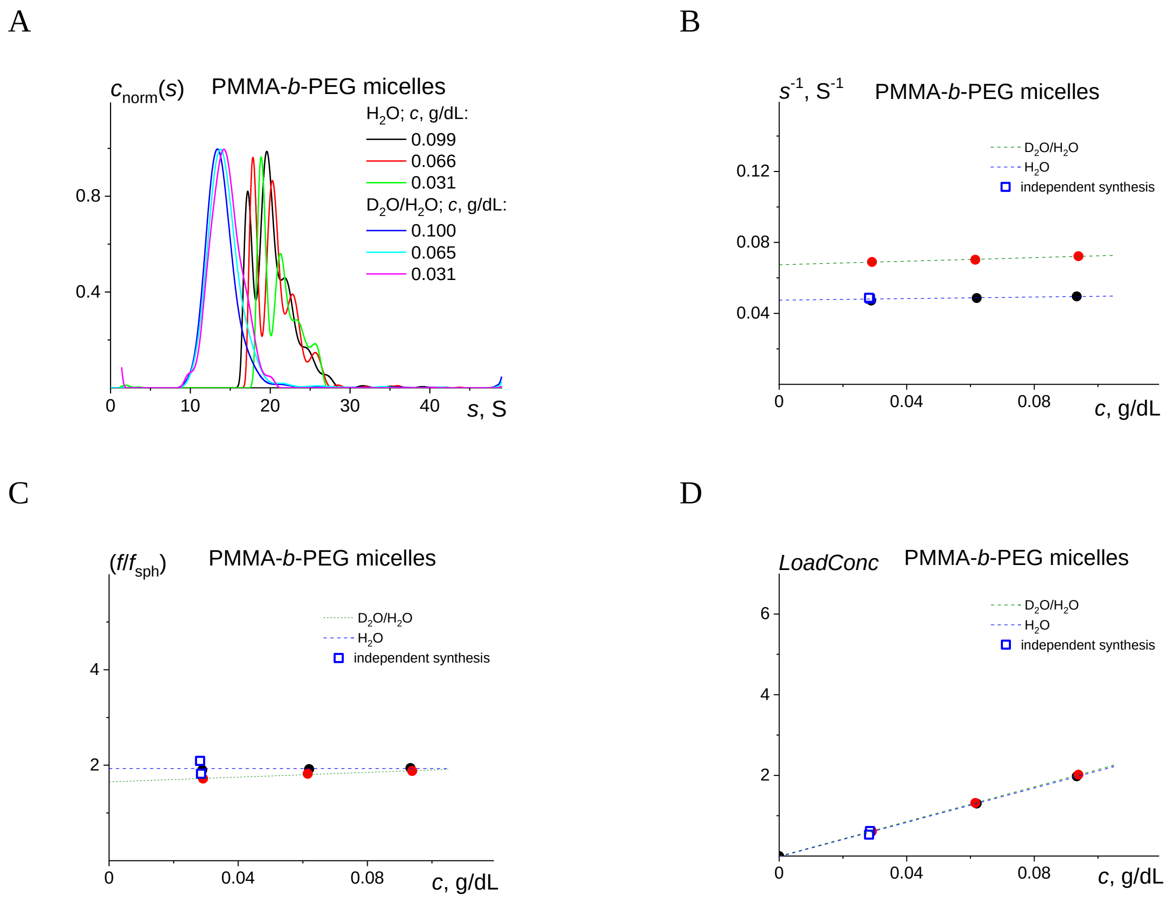


**Figure S11.** Summary of the velocity sedimentation experiment with PMMA-*b*-PEG block copolymer micelles in $H_2O$ and $D_2O/H_2O$ (50/50 vol.) mixture at 25 °C. (A) The normalized *c*(*s*) distributions on sedimentation coefficient *s* resolved with Sedfit 'continuous c(s) distribution' at three different concentrations in both solvents. (B) Concentration dependences of sedimentation coefficients. (C) Concentration dependences of the frictional ratios ($f/f_{sph}$). (D) Determination of the refractive index increment d*n*/d*c*.

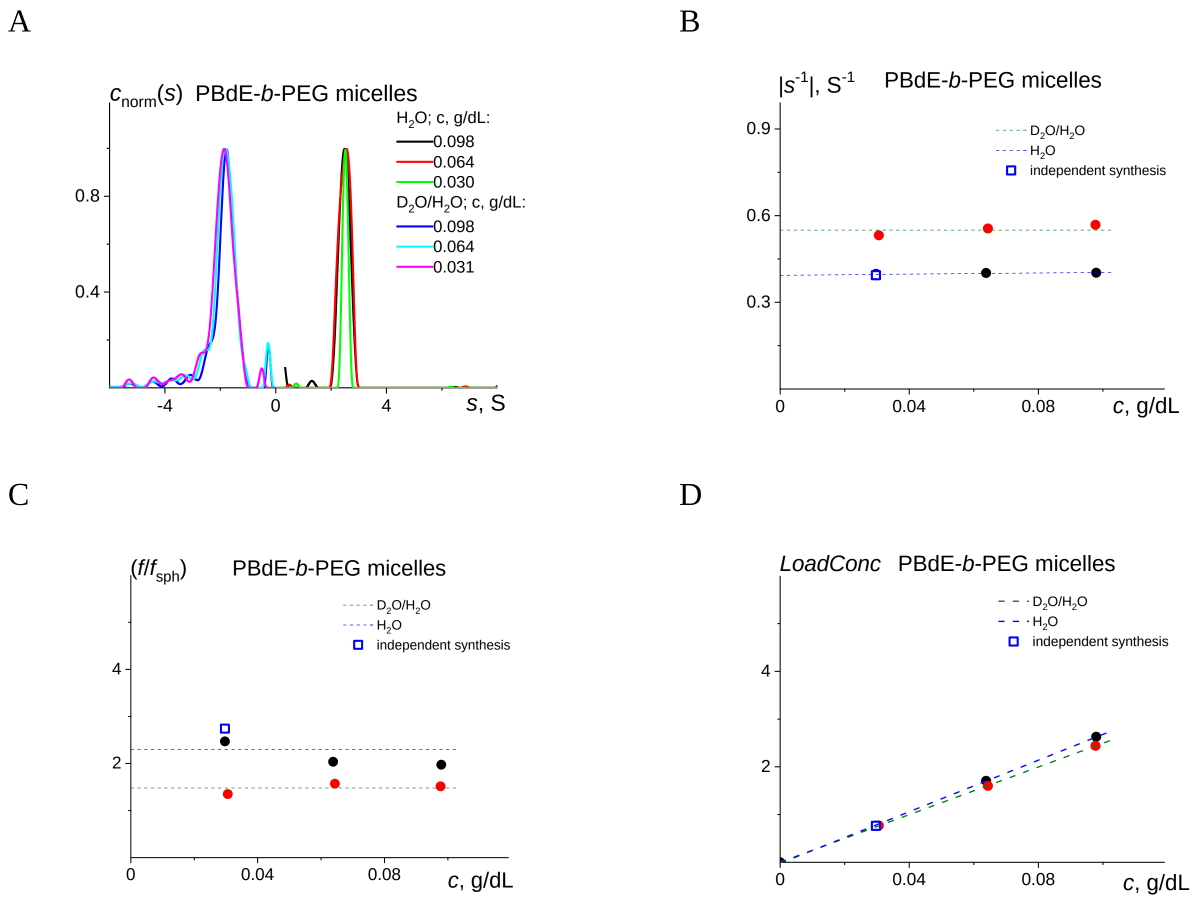


**Figure S12.** Summary of the velocity sedimentation experiment with PBd-*b*-PEG block copolymer micelles in $H_2O$ and $D_2O/H_2O$ (50/50 vol.) mixture at 25 °C. (A) The normalized $c(s)$ distributions on sedimentation coefficient $s$ resolved with Sedfit ‘continuous c(s) distribution’ at three different concentrations in both solvents. (B) Concentration dependences of sedimentation coefficients. (C) Concentration dependences of the frictional ratios ($f/f_{sph}$). (D) Determination of the refractive index increment d$n$/d$c$.

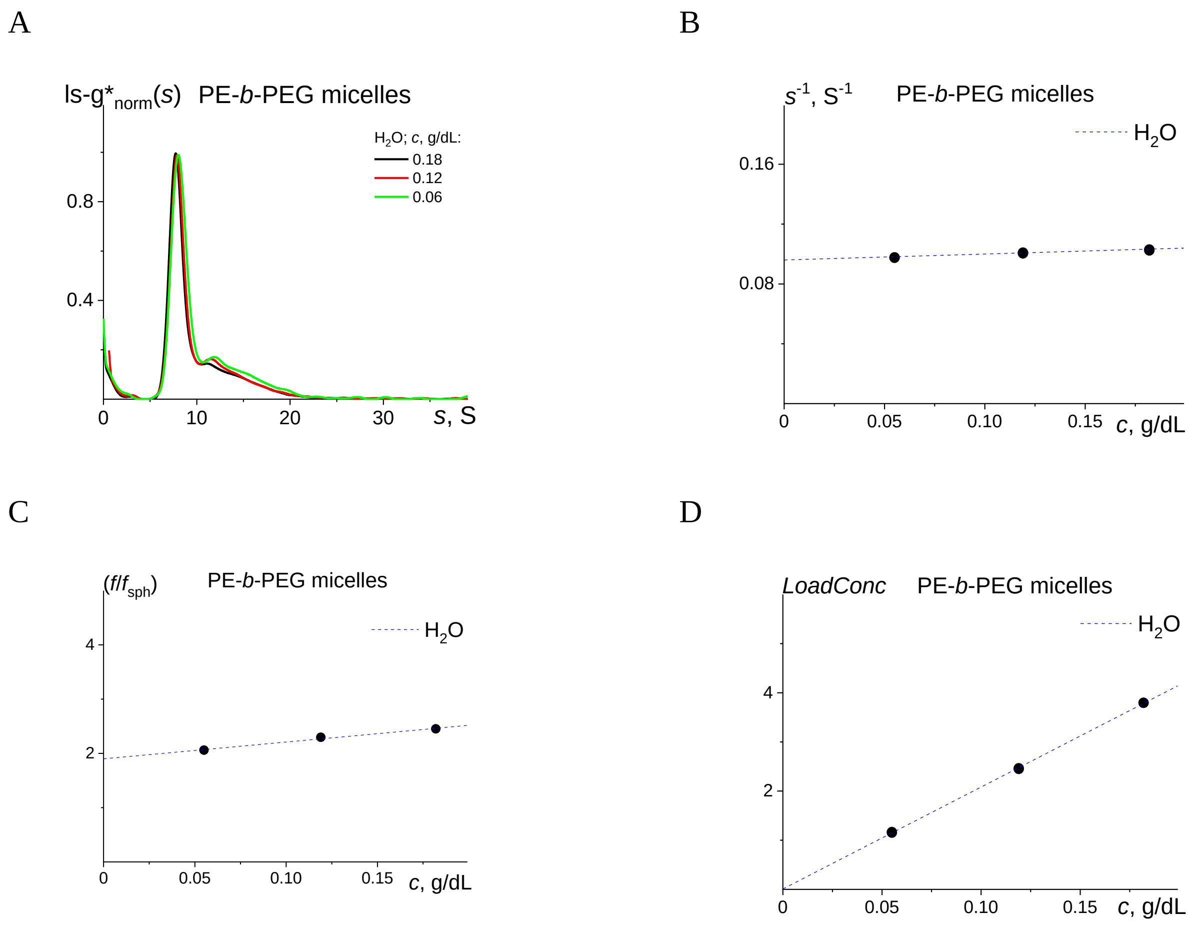


**Figure S13.** Summary of the velocity sedimentation experiment with PE-*b*-PEG block copolymer micelles in $H_2O$ at 25 °C. (A) The normalized ls-g*(*s*) distributions on sedimentation coefficient *s* resolved with Sedfit 'least square direct boundary approximation model' at three different concentrations. (B) Concentration dependences of sedimentation coefficients. (C) Concentration dependences of the frictional ratios ($f/f_{sph}$). (D) Determination of the refractive index increment d*n*/d*c*.

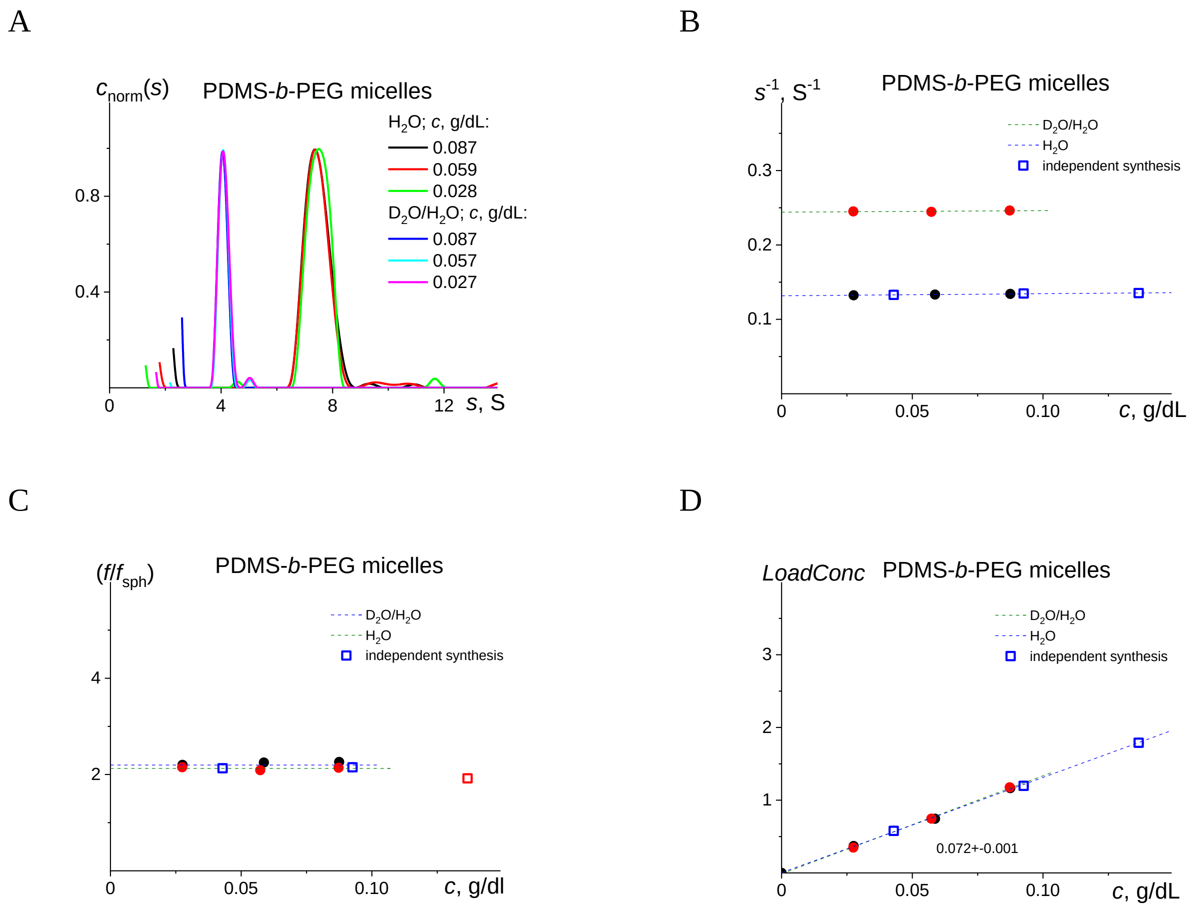


**Figure S14.** Summary of the velocity sedimentation experiment with PDMS-*b*-PEG block copolymer micelles in $H_2O$ and $D_2O/H_2O$ (50/50 vol.) mixture at 25 °C. (A) The normalized *c*(*s*) distributions on sedimentation coefficient *s* resolved with Sedfit 'continuous c(s) distribution' at three different concentrations in both solvents. (B) Concentration dependences of sedimentation coefficients. (C) Concentration dependences of the frictional ratios ($f/f_{sph}$). (D) Determination of the refractive index increment d*n*/d*c*.

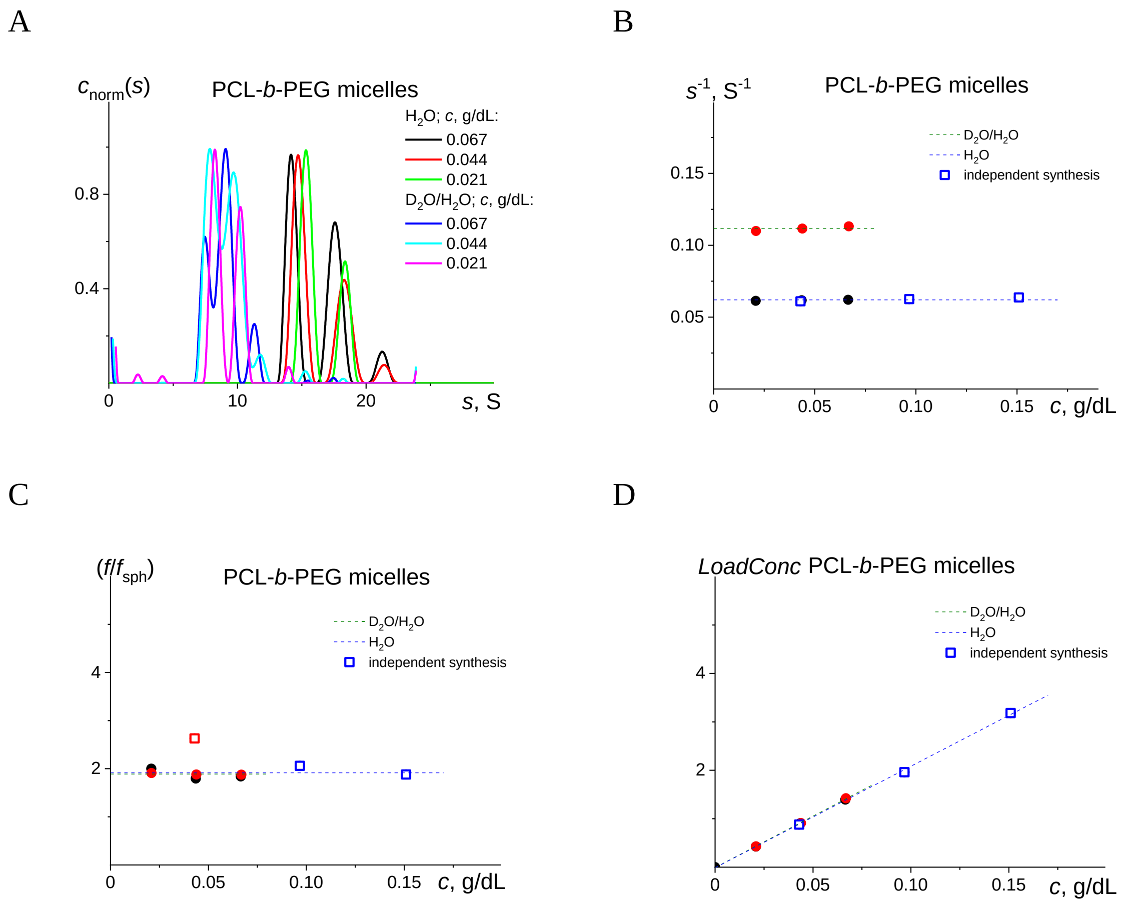


**Figure S15.** Summary of the velocity sedimentation experiment with PCL-*b*-PEG block copolymer micelles in $H_2O$ and $D_2O/H_2O$ (50/50 vol.) mixture at 25 °C. (A) The normalized *c*(*s*) distributions on sedimentation coefficient *s* resolved with Sedfit ‘continuous c(s) distribution’ at three different concentrations in both solvents. (B) Concentration dependences of sedimentation coefficients. (C) Concentration dependences of the frictional ratios ($f/f_{sph}$). (D) Determination of the refractive index increment d*n*/d*c*.
